# Observation on the therapeutic effect of probiotics on early oral feeding in the treatment of severe acute pancreatitis

**DOI:** 10.3389/fmed.2024.1492108

**Published:** 2024-12-03

**Authors:** Yanan Zhao, Rui Zhang, Shuling Wang, Chunchun Yang, Yang Wang, Hongyun Fan, Mingyue Yang

**Affiliations:** Department of Gastroenterology, First Hospital of Hebei Medical University, Shijiazhuang, Hebei, China

**Keywords:** pancreatitis, probiotics, enteral nutrition, inflammation, treatment outcome

## Abstract

**Objectives:**

To evaluate the clinical efficacy of probiotics and early oral feeding in patients with severe acute pancreatitis.

**Methods:**

A prospective, randomized, controlled trial was conducted involving 66 patients, who were randomly divided into a control group (*n* = 32) receiving standard enteral nutrition and an observation group (*n* = 34) receiving additional Bifidobacterium quadruplex live bacterial tablets. Serum inflammatory markers, including white blood cells (WBC), interleukin-6 (IL-6), tumor necrosis factor-*α* (TNF-α), and C-reactive protein (CRP), were measured on days 1, 3, and 7 post-admission. Abdominal pain scores, the computed tomography severity index (CTSI), and the Bedside Index for Severity in Acute Pancreatitis (BISAP) scores were also assessed. Additionally, defecation time and the total duration of hospitalization were compared between the two groups.

**Results:**

Inflammatory markers declined in all groups by the third day post-admission, with the observation group exhibiting a significantly greater reduction compared to the control group (*p* < 0.05). Similarly, from the first day to the third day, both groups experienced a decrease in abdominal pain scores, CTSI, and BISAP scores, with the observation group showing a significantly more pronounced decrease in BISAP scores compared to the control group (*p* < 0.05). By the seventh day of admission, inflammatory markers continued to decline in all groups compared to the third day, except for TNF-*α* levels, and the observation group demonstrated a significantly greater decrease compared to the control group (*p* < 0.05). Abdominal pain scores, CTSI, and BISAP scores also decreased further in both groups compared to the third day, with the observation group again showing a significantly greater improvement than the control group (*p* < 0.05). Additionally, the observation group had a significantly shorter time to bowel movement resumption (38.23 ± 2.31 h vs. 43.43 ± 2.75 h, *p* = 0.013) and total hospital stay compared to the control group (10.97 ± 0.35 days vs. 13.40 ± 0.50 days, *p* < 0.001).

**Conclusion:**

Early oral ingestion combined with probiotics can reduce the levels of inflammatory factors, improve abdominal pain symptoms, alleviate pancreatic edema and shorten defecation time and hospital stay in patients with severe acute pancreatitis.

## Introduction

Acute pancreatitis (AP) is characterized by its sudden onset, rapid progression, multiple complications, and high mortality rate. Its main features include self-digestion of the pancreas, local inflammatory reactions, among others. Severe acute pancreatitis (SAP), accounting for approximately 5 to 10% of AP cases, often leads to persistent organ failure in patients (1). Additionally, SAP patients are prone to developing secondary infections, shock, and peritonitis, among other complications. The disease progresses rapidly, with a high mortality rate, posing significant challenges in clinical management. Therefore, the correct clinical treatment methods are extremely important. Most physicians currently advocate early enteral nutrition under patient tolerance, in order to protect the intestinal mucosal barrier function, reduce bacterial translocation, and lower plasma endotoxin levels in SAP patients, thereby reducing patients mortality (2).

Research has found that adding probiotics, especially bifidobacteria, while providing enteral nutrition can inhibit the proliferation of harmful bacterial communities, reduce inflammatory reactions, increase the intestinal mucosal barrier, and promote the recovery of gastrointestinal function (3). While previous research has extensively investigated the pathological changes in critically ill patients at a basic research level, particularly regarding the impact of probiotics, there remains a scarcity of reports on the effects of probiotics on inflammatory cytokine levels, improvement in clinical symptoms, especially in terms of bowel movement and length of hospital stay in AP patients at the clinical level. Therefore, the aim of this study is to observe the efficacy of probiotics in the early enteral nutrition process of SAP patients, providing specific references for clinical treatment.

## Materials and methods

### General information

A total of 66 SAP patients admitted to our hospital from January 2023 to September 2023 were selected as the study subjects. The diagnostic criteria for acute pancreatitis include the following three items: (1) persistent upper abdominal pain; (2) The concentration of serum amylase and/or lipase is three times higher than the upper limit of normal; (3) The results of abdominal imaging examination showed that it was consistent with the imaging changes of acute pancreatitis. If two of the above three criteria are met, it can be diagnosed as acute pancreatitis. According to the determinant-based classification, acute severe pancreatitis refers to the occurrence of organ dysfunction or infectious necrosis for more than 48 h. The inclusion criteria were: ① meeting the diagnostic criteria for severe acute pancreatitis in the “Guidelines for the Diagnosis and Treatment of Acute Pancreatitis (2021)” (4); ② Aged between 18 and 70 years old; ③ Clear awareness and good communication skills; ④ Those who voluntarily participate in this study and have good compliance. Exclusion criteria: ① Patients with concurrent biliary obstruction, intestinal paralysis, intestinal fistula, intestinal perforation, and other diseases; ② Individuals with immune system diseases; ③ Individuals with contraindications for early enteral nutrition support treatment: The patients have shock, complete intestinal obstruction, digestive tract perforation, gastrointestinal hemorrhage, severe abdominal pain and bloating; ④ Pregnant and lactating women. They were divided into a control group (32 cases) and an observation group (34 cases) using a random number table method.

This study has been approved by the hospital’s ethics committee (No. 20220579) and obtained informed consent from the patients.

### Treatment plan

After admission, both groups of patients were treated with fasting water, inhibiting pancreatic enzyme secretion (Somatostatin for injection 250ug/h continuous pumping point), acid suppression (Esomeprazole Sodium for Injection 20 mg intravenous drip once a day), analgesia (When the patient has obvious abdominal pain, administer 5 mg of Dizocin injection to the patient’s muscles), fluid replacement (Within the first 12–24 h, replenish 250-500 mL of isotonic crystal fluid per hour, and repeat assessment every 6 h), correcting electrolyte disorders (Supplement sodium, potassium, chloride, calcium, phosphorus, magnesium), regulating blood sugar, and promoting gastrointestinal motility (When the patient’s abdominal pain is tolerable, they should get out of bed and move around appropriately). The control group began to eat by mouth 24 h after admission and drink thin rice soup (50 mL each time, 6 times in total) on the first day. If the patients could basically tolerate it, they could eat semi liquid food such as porridge, noodle soup, thin noodles, vegetable paste (100 mL each time, 3 times in total) on the second to sixth day. The intake was properly increased according to the intestinal function. If there was no discomfort, they could eat steamed bread, rice, egg soup and other conventional low-fat solid food on the seventh day. On the first day of admission, the experimental group received an additional 1.5 g/day of oral Bifidobacterium quadruplex live bacterial tablets (Hangzhou Yuanda Biopharmaceutical Co., Ltd. National Drug Approval No. S20060010) on the basis of the control group. Patients’ adverse reactions were observed through survey questionnaires. If all patients experience mild abdominal pain, bloating, and other symptoms that can be tolerated, no intervention will be given temporarily. If abdominal pain and bloating are clearly intolerable, they will stop eating and eat again 24 h later. If the patients still cannot tolerate it, withdraw from the trial.

### Detection indicators

To evaluate and compare the concentrations of C-reactive protein (CRP), white blood cells (WBC), interleukin-6 (IL-6), and tumor necrosis factor alpha (TNF-*α*) between the two groups, blood samples were collected on the 1st, 3rd, and 7th days following patient admission. A 5 mL sample of fasting venous blood was obtained from each participant, and the serum was separated by centrifugation. White blood cell counts were determined using flow cytometry, while the concentrations of CRP, IL-6, and TNF-*α* were quantified via enzyme-linked immunosorbent assay (ELISA). Additionally, the abdominal pain scores (assessed using the Visual Analog Scale [VAS]) (5), the CT severity index (CTSI) (6), and the Bedside Index for Severity in Acute Pancreatitis (BISAP) (7) were compared between the two groups on the 1st, 3rd, and 7th days post-admission. The time to first defecation and the total length of hospital stay were also compared at the time of discharge.

### Sample size calculation

The sample size for this study was calculated based on the primary outcome, which was the difference in serum concentrations of C-reactive protein (CRP) between the control and observation groups. A preliminary study or data from similar studies was used to estimate the effect size and standard deviation for CRP levels. Assuming a two-sided significance level (*α*) of 0.05, a power (1-*β*) of 0.80, and an effect size of 0.7, a minimum of 30 patients per group was required. To account for potential dropouts or exclusions, the sample size was increased by approximately 10%, resulting in a final total of 66 patients (32 in the control group and 34 in the observation group). The sample size calculation was performed using G*Power 3.1 software.

### Statistical analysis

The obtained data was analyzed using SPSS 22.0 software, and the measurement data was expressed as mean ± standard deviation (^−^x ± s) using t-test. The counting data was expressed as rate (%) using *χ* 2 tests, with *p* < 0.05 as the difference with statistical significance.

## Results

### General information

The control group consisted of 10 cases of hyperlipidemia, 18 cases of cholelithiasis, and 4 cases of alcoholism. There were 11 cases of diabetes, 8 cases of hypertension and 4 cases of functional gastrointestinal diseases. There were 21 males and 11 females, aged 20–69 years, with an average age of 44.65 ± 2.51 years. The BISAP score at admission was 3.34 ± 0.19 points; in the observation group, there were 11 cases of hyperlipidemia, 20 cases of cholelithiasis, and 3 cases of alcoholism. There were 12 cases of diabetes, 6 cases of hypertension and 6 cases of functional gastrointestinal diseases. There were 20 males and 14 females, aged 22–68 years, with an average age of 45.40 ± 2.28 years. The BISAP score at admission was 2.96 ± 0.20. There was no statistically significant difference in general information and BISAP scores between the two groups of patients (*p* > 0.05), indicating comparability. Both groups of patients did not have endothelial vascular diseases, metabolic syndrome, ulcerative colitis, Crohn’s disease, or other diseases.

### Comparison of inflammatory indicators

On the first day of admission, there was no statistically significant difference in the concentration of CRP, WBC, IL-6, TNF- *α* between the observation group and the control group. On the third day of admission, inflammation indicators in all groups decreased compared to the first day, and the observation group showed a more significant decrease compared to the control group (*p* < 0.05). On the 7th day of admission, inflammation indicators in all groups decreased compared to the 3rd day, except for TNF- *α*, and the observation group showed a more significant decrease compared to the control group (*p* < 0.05). The results are shown in Table 1.

**Table 1:** Changes in inflammatory indicators at different times in two groups.

Group	WBC (10^9/L)	CRP (mg/L)	IL-6 (pg/ml)	TNF-*α* (pg/ml)
The control group (*n* = 32)				
Day1	12.58 ± 3.43	156.88 ± 18.24	641.67 ± 90.09	13.10 ± 1.45
Day3	10.03 ± 3.10^#^	121.70 ± 10.26^#^	415.36 ± 69.12^#^	7.91 ± 0.83^#^
Day7	7.72 ± 2.56^*^	68.22 ± 6.19^*^	94.77 ± 14.06^*^	3.68 ± 0.17^*^
The observation group (*n* = 34)				
Day1	12.61 ± 3.51	151.61 ± 17.07	631.91 ± 81.63	12.65 ± 1.28
Day3	8.94 ± 2.89^#@^	103.45 ± 7.84^#@^	365.73 ± 63.05^#@^	7.93 ± 0.79^#@^
Day7	6.81 ± 2.40^*@^	49.44 ± 5.43^*@^	53.19 ± 11.51^*@^	3.81 ± 0.22^*^

^#^Compared with the same group on the first day, *p* < 0.05; ^*^Compared with the same group on the 3rd day, *p* < 0.05; ^@^Compared with the control group on the same day, *p* < 0.05; WBC, white blood cells; CRP, C-reactive protein; IL-6, interleukin-6; TNF-*α*, tumor necrosis factor-α.

### Comparison of clinical scores

On the first day of admission, there was no statistically significant difference in abdominal pain score (VAS scoring method), CTSI, and BISAP between the two groups of patients; On the third day of admission, the above scores of both groups of patients decreased compared to the first day, with the BISAP observation group showing a more significant decrease compared to the control group (*p* < 0.05); On the 7th day of admission, the above scores of both groups of patients decreased compared to the 3rd day, and the observation group showed a more significant decrease compared to the control group (*p* < 0.05). The results are shown in Table 2.

**Table 2:** Changes in abdominal pain scores, CTSI, and BISAP between two groups at different times.

Group	Abdominal pain score	CTSI	BISAP
The control group (*n* = 32)			
Day1	7.25 ± 0.17	5.31 ± 0.43	3.59 ± 0.16
Day3	4.46 ± 0.22^#^	4.03 ± 0.34^#^	2.81 ± 0.15
Day7	2.62 ± 0.17^*^	2.40 ± 0.20^*^	1.59 ± 0.08
The observation group (*n* = 34)			
Day1	7.26 ± 0.15	5.38 ± 0.40	3.61 ± 0.16
Day3	4.44 ± 0.20^#^	4.00 ± 0.31^#^	2.61 ± 0.12^#@^
Day7	2.05 ± 0.13^*@^	1.97 ± 0.13^*@^	1.26 ± 0.12^*@^

^#^Compared with the same group on the first day, *p* < 0.05; ^*^compared with the same group on the 3rd day, *p* < 0.05; ^@^compared with the control group on the same day, *p* < 0.05; CTSI, computed tomography severity index; BISAP, bedside index for severity in acute pancreatitis.

### Comparison of defecation time and total hospitalization time

Compared with the control group, the observation group showed a decrease in defecation time and total hospitalization time (*p* < 0.05). The results are shown in Table 3.

**Table 3:** Comparison of defecation time and total hospitalization time between two groups.

Group	Defecating time (h)	Total hospitalization time (d)
The control group (*n* = 32)	43.43 ± 2.75	13.40 ± 0.50
The observation group (*n* = 34)	38.23 ± 2.31	10.97 ± 0.35
*t*	2.55	3.95
*p* value	0.013	<0.001

### Adverse reactions

There are two patients in the control group experienced mild nausea and abdominal pain, both of which were tolerable. No patients were excluded from the study.

## Discussion

SAP is a critical gastrointestinal disease, with most SAP patients succumbing to infectious complications. Research has shown that intestinal bacterial translocation occurs in the abdomen of SAP patients, indicating that infectious complications of pancreatic infection are caused by intestinal bacterial translocation (8, 9). Therefore, in order to prevent intestinal bacterial translocation and improve intestinal function in SAP patients, early enteral nutrition is commonly used in clinical practice for intervention.

The traditional view is that patients with AP should undergo fasting and parenteral nutrition to reduce the impact on the pancreas by reducing its own digestion and the resecretion of tissue damaging enzymes. However, according to relevant studies, unlike normal pancreatic secretion physiological processes, pancreatic acinar secretion function in SAP patients is significantly inhibited, and most patients still have pancreatic exocrine dysfunction even during the recovery period (10–12). This pathological feature also serves as the theoretical basis for the safety of oral feeding in the clinical treatment of SAP. Eating orally can induce the secretion of intestinal hormones, which can directly act on the pancreas and effectively suppress inflammatory reactions. Some clinical studies and animal experiments have also found that enteral nutrition can promote intestinal barrier repair by affecting intestinal permeability, immune cell activity, and bacterial translocation, thereby promoting the recovery of SAP and reducing mortality (13). During the initial oral intake, abdominal discomfort symptoms such as abdominal pain, nausea, and vomiting may occur, but it is still recommended to start enteral nutrition within 48 or even 24 h of admission when the patient can tolerate it (14).

Studies have shown that compared to healthy individuals, the feces of AP patients have significantly higher amounts of *Escherichia coli* and Enterococcus, while bifidobacteria are significantly reduced (15); Moreover, the overall structure and diversity of dominant gut microbiota in SAP patients show more significant changes. When the gut microbiota is imbalanced, the first to be disrupted are the intestinal inflammatory and immune responses (16). Tan et al. (17) found that the content of serum interleukin-6 was positively correlated with the abundance of *Escherichia coli* and Enterococcus, negatively correlated with the abundance of Bifidobacterium, and positively correlated with the abundance of plasma endotoxin and Enterococcus. This discovery suggests that the inflammatory response is largely related to dysbiosis of the gut microbiota, which can also affect the mechanical barrier of the intestinal mucosa. The increased permeability of intestinal mucosa can lead to bacterial translocation, pancreatic tissue necrosis and infection, and even multiple organ dysfunction syndrome. Therefore, it is believed that there is a significant correlation between patients with acute pancreatitis and their gut microbiota changes (18).

In some early guidelines, empirical use of local and intestinal antibiotics (SBD) is recommended to reduce bacterial overgrowth and reduce organ infection and necrosis. However, due to the risk of bacterial resistance and fungal infection, SDB has not been widely implemented (19, 20). A meta-analysis designed by Wittau et al. on the prophylactic use of antibiotics in SAP patients showed that prophylactic use of antibiotics did not reduce the risk of infectious pancreatic necrosis (21). Multiple studies have shown that probiotics can reduce inflammation and infection related complications in SAP patients (22, 23). The main mechanism of probiotics in treating SAP is: (1) Promote the restoration of gut microbiota diversity; (2) Change intestinal pH and inhibit the growth of harmful bacteria; (3) Increase the number of intestinal epithelial cells and enhance the intestinal barrier function; (4) Reduce visceral pain (24). Studies have shown that adding probiotics during early enteral nutrition can improve patients’ nutritional status, increase the content of beneficial bacteria in the intestine and reduce the levels of D-lactate, diamine oxidase, and plasma endotoxin (25, 26).

The probiotics used in this study are Bifidobacterium quadruplex live bacterial tablets. It is a composite preparation composed of infant Bifidobacterium, *Bacillus cereus*, *Enterococcus faecalis*, and *Lactobacillus acidophilus*, which has shown good efficacy in treating constipation, diarrhea, functional dyspepsia, and non-alcoholic fatty liver disease in clinical practice (27, 28). The probiotic quadruple combination of Lactobacillus can enhance patient immunity, repair intestinal mucosal barrier function, and regulate imbalances in the intestinal microbiota (29). Early enteral nutrition can help rapidly restore intestinal function by promoting nutrient absorption, repairing intestinal mucosal cell structure, reducing intestinal infections, and preventing bacterial translocation. Therefore, the combination of the probiotic quadruple combination of Lactobacillus with early enteral nutrition may improve the therapeutic effects of severe AP in clinical practice.

During the onset of acute pancreatitis, necrosis of acinar cells increases the activity of pancreatic trypsin in pancreatic cells, releasing various inflammatory cytokines and activating the inflammatory cascade reaction, and white blood cells will increase. In addition, pro-inflammatory cytokines (TNF, IL-1, 6, and 8), arachidonic acid metabolites (prostaglandins, platelet activating factors, and leukotrienes), and CRP will also be released. These inflammatory factors can lead to pancreatic injury and can predict complications and prognosis (30), the level of inflammatory markers can evaluate the severity of pancreatitis. This study conducted assays on the changes of white blood cells, CRP, IL-6, and TNF- *α* to evaluate the inflammatory changes in patients. Oral administration of probiotics in the observation group can reduce the levels of inflammatory factors, improve symptoms and shorten defecation time and hospital stay. These reports can serve as a reference for clinical treatment of SAP.

This study has several limitations. Firstly, our results may not be applicable to all AP patients on account of only including some SAP patients with a small sample size; Secondly, the results of this study are not applicable to all probiotics, because we only used Bifidobacterium tetravalent live bacteria. At present, there are various types of probiotics, and clinical treatment plans and individual differences may lead to differences or inconsistencies with our research results. Thirdly, studies have shown that the use of probiotics may lead to adverse reactions such as gastrointestinal bloating, so individualized treatment is necessary during clinical use (31).

## Conclusion

In conclusion, the combination of early oral intake with probiotics can effectively reduce inflammatory cytokine levels, improve clinical symptoms, and shorten the time to bowel movements and hospital stay in patients with severe acute pancreatitis. These findings provide new insights and strategies for the clinical treatment of severe acute pancreatitis. Furthermore, further exploration is needed to elucidate the precise mechanisms of action of probiotics and their safety and efficacy in different populations, aiming to provide more personalized and effective treatment options for patients with severe acute pancreatitis.

## Data Availability

The original contributions presented in the study are included in the article/supplementary material, further inquiries can be directed to the corresponding author.
